# Prevention of intradialytic hypotensive episodes: is setraline an effective pharmacological approach?

**DOI:** 10.1590/2175-8239-JBN-2019-0175

**Published:** 2019-12-02

**Authors:** Panagiotis I. Georgianos, Rajiv Agarwal

**Affiliations:** 1 Aristotle University of Thessaloniki, AHEPA Hospital, 1st Department of Medicine, Section of Nephrology and Hypertension, Thessaloniki, Greece.; 2 Indiana University, School of Medicine, Department of Medicine, Division of Nephrology and Richard L. Roudebush Veterans Administration Medical Center, Indianapolis, IN, USA.

Intradialytic hypotension (IDH) is an important clinical complication of hemodialysis for patients, physicians, and dialysis technicians and nurses. Besides causing uncomfortable symptoms (i.e. cramps or postdialysis fatigue), IDH is associated with an increased risk for vascular access thrombosis,^1^ inadequate delivery of dialysis, and future cardiovascular morbidity and mortality.^2^ Whether these risks are causal remains unknown. The European Best Practice Guidelines (EBPG) define IDH as a drop in systolic blood pressure (SBP) ≥ 20 mmHg or decrease in mean arterial pressure by 10 mmHg, being accompanied by symptoms of end-organ ischemia and necessitating nursing interventions.^3^ This clinically relevant and rigorous definition is difficult to apply since many of the elements of the definitions are missing from administrative databases. Perhaps more troubling is the fact that this definition has not been consistently adopted in clinical research. Due to substantial heterogeneity in the way that IDH has been defined across studies, the exact burden of this complication remains unknown.^4^ In a recent meta-analysis with 5 studies that included 1694 patients, the prevalence of hemodialysis sessions complicated by IDH as defined by the EBPG criteria was 10.1% (range 5.0 to 30.8%); among 203,768 patients in 5 other studies, the nadir SBP < 90 mmHg had a prevalence of 11.6% (range 6.7% to 17.2%).^5^ However, the proportion of patients with frequent IDH varied from study to study because of varying definitions of frequent IDH.^5^ Definitions of frequent IDH ranged from at least one session in 3 months to 33% of the sessions over 3 months.

Although the complications of IDH are well-recognized, the close relationship between IDH and interdialytic hypertension is underappreciated. This is because recurrent episodes of IDH and management strategies commonly applied (i.e., premature cessation of dialysis, hypertonic saline infusion, increase in dialysate sodium concentrations) act as barriers against dry-weight achievement and predispose these patients to risks arising from volume overload leading to interdialytic hypertension.^6^ Thus, the scenario of a patient presenting recurrent episodes of IDH to be concomitantly on a volume-expanded state with uncontrolled blood pressure (BP) outside of dialysis is not rare.

In this issue of the *Brazilian Journal of Nephrology,* Molin C. et al.^7^ report a “double-blind, placebo-controlled, cross-over” study testing the efficacy of the selective serotonin reuptake inhibitor sertraline in preventing IDH. According to the inclusion/exclusion criteria, participants had IDH complication (defined as a drop of ≥ 30 mmHg in SBP within dialysis or predialysis SBP ≤ 100 mmHg accompanied by symptoms requiring nursing interventions) in at least 50% of dialysis sessions over a 3-month period prior to study enrollment. In addition, patients having SBP < 90 mmHg during dialysis or diastolic BP < 40 mmHg could qualify. Finally, any patient requiring intervention regardless of the BP criteria could be included in this study. Participants entered an initial 6-week placebo phase and then were switched to active-treatment with sertraline for another 6 weeks.^7^ Among 55 patients screened, 18 (32%) met the prespecified inclusion/exclusion criteria and 16 patients completed the trial. The occurrence of IDH episodes did not significantly differ between the sertraline and placebo phases. However, the risk of reporting adverse intradialytic symptoms was 42% higher with placebo [hazard ratio (HR): 1.42; 95% confidence interval (CI): 1.02-2.02].^7^ This effect was accompanied by 59% higher likelihood for nursing intradialytic interventions during the placebo phase (HR: 1.59; 95% CI: 1.03-2.48).^7^

Although interventional studies are important and the efforts of Molin et al. are worthy of appreciation, a number of lessons can be taken from this report. This study is not double-blind because the investigators were aware of the drug assignment, therefore it has a single-blind, placebo run-in design. Second, a cross-over design conventionally means that patients on placebo switch to drug and those on drug switch to placebo; no cross-over took place in that study, which had simply a before-and-after study design. The sample size estimation assuming a 90% effect size (reduction in IDH from 50% to 5%) required 30 patients according to the authors, yet 16 were included. Thus, the study is underpowered despite evaluating a large effect size. The 95% CIs reported for intradialytic BP measurements are not credible given the large standard deviations. Finally, the Kaplan-Meier curves displayed do not agree with the p value of the log-rank tests reported. Thus, on many counts, the study is squarely negative.

Sertraline has been tested as a pharmacological approach for orthostatic hypotension, neurocardiogenic syncope, or IDH. The main mechanism through which this agent exerts a favorable hemodynamic action is the amelioration of the paradoxical sympathetic withdrawal induced by a sudden surge of serotonin in the central nervous system. In accordance with the above results of the Molin study,^7^ earlier interventional studies enrolling hemodialysis patients with IDH failed to show improvement in the incidence of IDH or cardiac output, central blood volume, and peripheral vascular resistance in response to therapy with sertraline.^8^ Other studies have associated sertraline administration with improvement in nadir intradialytic SBP or with fewer nursing interventions required for IDH.^9^ However, these studies suffer from certain methodological limitations and, mainly, they lack a proper adjudication of intradialytic hypotensive episodes.^9^ In our interpretation, the currently available evidence cannot demonstrate a clear hemodynamic benefit of sertraline in preventing IDH. This agent, however, appears to exert a favorable anti-depressant action that is accompanied by less frequent reporting of uncomfortable intradialytic symptoms. Whether this anti-depressant effect is translated into a long-term benefit on domains of health-related quality of life remains to be elucidated in larger studies with longer follow-up periods.

The question that remains crucial is how can we prevent the occurrence of IDH and how can we improve patient outcomes. A summary of measures that may be beneficial is provided in Table 1. In our practice, the first-line management of IDH incorporates a careful assessment of dry-weight.^4^^,^ 6 Although increasing dry-weight is commonly used as an initial approach to reduce the necessity for aggressive ultrafiltration rates, this decision should be carefully balanced against its potential risks. Since routine BP recordings taken within the dialysis unit cannot accurately detect the presence of interdialytic hypertension,^10^ we recommend home BP monitoring in order to assess BP control in the interdialytic period. In patients experiencing frequent episodes of IDH but their out-of-dialysis BP is inadequately controlled or manifest other signs of volume excess, increasing dry-weight is not a quick fix for this complex condition. In this rather common scenario, dietary sodium restriction, avoidance of intradialytic sodium gain through individualized prescription of dialysate sodium concentrations, and extending the duration of dialysis to at least 4 hours/session are more effective strategies to limit interdialytic weight gain, preserve the hemodynamic stability with ultrafiltration, and improve the overall BP control.^4^^,^ 6 A recent analysis of 10,250 participants in phase 4 of the Dialysis Outcomes and Practice Patterns Study (DOPPS) support that this therapeutic approach is associated with improvement in clinical outcomes^11^. A facility practice of timely dry-weight assessment was associated with 28% reduced risk of cardiovascular mortality (HR: 0.72; 95% CI: 0.55-0.95).^11^ By contrast, routine use of sodium profiling as a measure against IDH (an approach that results in positive net intradialytic sodium balance) was associated with 34% higher risk of cardiovascular mortality (HR: 1.34; 95% CI: 1.04-1.73).^11^ Nonetheless, we believe that that the dialysis prescription needs to be individualized.

**Table 1:** Interventions that may be beneficial in preventing intradialytic hypotension

Intervention
1) Dry weight assessment
2) Dietary sodium restriction
3) Individualized dialysate sodium prescription
4) Ensure the adequate duration of dialysis (at least 4 hours 3 times per week)
5) Avoid eating during dialysis
6) Cool dialysate
7) Optimize antihypertensive regimen (i.e., discontinue short-acting BP-lowering medications prior to dialysis)
8) Ultrafiltration modeling
9) Consider to increase the dialysis frequency (i.e., short-daily dialysis)

In conclusion, IDH is a common complication that limits the adequacy of dialysis and worsens the patient outcomes. In our practice, we base the management of IDH on the adequate assessment of dry weight and sodium restriction. Based on the available evidence, the use of sertraline or other agents (i.e. carnitine, midodrine) appears to be of no value in preventing IDH.
